# 514. Dynamics of Tissue Regeneration After Deep Dermal Burns

**DOI:** 10.1093/jbcr/irag033.173

**Published:** 2026-04-06

**Authors:** Manuel Prevedel, Anna-Lisa Pignet, Martin Funk, Ives Bernardelli de Mattos, Tobias Madl, Johanna Einsiedler, David Hahn, Anita Eberl, Andrzej Hecker, Thomas Birngruber, Lars-Peter Kamolz, Petra Kotzbeck, Elisabeth Hofmann

**Affiliations:** COREMED – Center for Regenerative and Precision Medicine, JOANNEUM RESEARCH Forschungsgesellschaft mbH, Graz, Austria, Graz, Steiermark; Division of Plastic, Aesthetic and Reconstructive Surgery, Department of Surgery, Medical University of Graz, Austria, Graz, Steiermark; EVOMEDIS GmbH, Graz, Steiermark; EVOMEDIS GmbH, Würzburg, Bayern; Medical University of Graz, Graz, Steiermark; Joanneum Research, Graz, Steiermark; COREMED – Center for Regenerative and Precision Medicine, JOANNEUM RESEARCH Forschungsgesellschaft mbH, Graz, Austria, Graz, Steiermark; HEALTH-Institute for Biomedicine and Health Sciences, JOANNEUM RESEARCH Forschungsgesellschaft mbH, Graz, Austria, Graz, Steiermark; Medical University of Graz, Department of Surgery, Division of Plastic, Aesthetic and Reconstructive Surgery, Graz, Steiermark; HEALTH-Institute for Biomedicine and Health Sciences, JOANNEUM RESEARCH Forschungsgesellschaft mbH, Graz, Austria, Graz, Steiermark; Division of Plastic, Aesthetic and Reconstructive Surgery, Department of Surgery, Medical University of Graz, Austria, Graz, Steiermark; COREMED – Center for Regenerative and Precision Medicine, JOANNEUM RESEARCH Forschungsgesellschaft mbH, Graz, Austria, Graz, Steiermark; COREMED – Center for Regenerative and Precision Medicine, JOANNEUM RESEARCH Forschungsgesellschaft mbH, Graz, Austria, Graz, Steiermark

## Abstract

**Introduction:**

Thermal injuries initiate a cellular cascade that extend beyond the site of injury, often resulting in significant tissue damage and systemic effects. Burn wounds are not static, as they undergo a complex progression in the first few days after injury, leading to deeper tissue involvement. This study investigates the dynamic progression of burn wounds, focusing on the histological features, inflammatory markers, and metabolic responses over time.

**Methods:**

Contact burns (80°C for 20 seconds) were induced on pigs (n = 4) at four different occasions. Biopsies and dermal interstitial fluid were obtained from wounds in different healing stages, i.e., 8 hours, 4, 11, and 21 days post burn, with unburned areas as controls. Histology, immunohistochemistry and gene expression analyses were performed on tissue biopsies. Metabolomics of tissue biopsies and the dermal interstitial fluid were assessed via NMR spectroscopy.

**Results:**

Histological analysis revealed deep dermal burns at 8 hours post-injury, progressing to full-thickness burns. Re-epithelialization began at 4 days, with complete epithelialization at 21 days. Inflammation peaked at 8 hours. Pro-inflammatory markers (IL-6, PGE2) and angiogenesis markers (VEGFA) showed a regressive dynamic. Metabolomics analysis revealed that myo-inositol, ketoleucine, and phosphorylcholine peaked at 8 hours but returned to baseline by day 4. Mannose decreased at 8 hours and 4 days, returning to baseline at 21 days.

**Conclusions:**

Our detailed analyses of burn wound dynamics in the present porcine in vivo study provide profound knowledge on the temporal dysregulation of key mediators such as cytokines and metabolites in the progress of burn wound healing.

**Applicability of Research to Practice:**

In the future a stage-resolved marker profile shall guide care decisions. Clinicians will be able to identify wounds at risk of full-thickness progression, time debridement and therapies to the shift from peak inflammation to emerging angiogenesis. Patient-level phenotyping (e.g., elevated early IL-6/PGE₂ or characteristic carbohydrate/amino-acid shifts) supports personalized selection of dressings, cadence of dressing changes, and timely escalation to advanced therapies.

**Funding for the study:**

Austrian Research Promotion Agency (FFG).

